# Thyroid Nodule Classification for Physician Decision Support Using Machine Learning-Evaluated Geometric and Morphological Features

**DOI:** 10.3390/s20216110

**Published:** 2020-10-27

**Authors:** Elmer Jeto Gomes Ataide, Nikhila Ponugoti, Alfredo Illanes, Simone Schenke, Michael Kreissl, Michael Friebe

**Affiliations:** 1 Clinic for Radiology and Nuclear medicine, Department of Nuclear Medicine, Otto-von-Guericke University Medical Faculty, 39120 Magdeburg, Germany; simone.schenke@med.ovgu.de (S.S.); michael.kreissl@med.ovgu.de (M.K.); 2 INKA-Application Driven Research, Otto-von-Guericke University Magdeburg, 39120 Magdeburg, Germany; nikhila.ponugoti@gmail.com (N.P.); alfredo.illanes@ovgu.med.de (A.I.); michael.friebe@ovgu.de (M.F.); 3 IDTM GmbH, 45657 Recklinghausen, Germany

**Keywords:** thyroid nodules, ultrasound imaging, TIRADS, feature extraction, machine learning, classification, computer aided diagnosis

## Abstract

The classification of thyroid nodules using ultrasound (US) imaging is done using the Thyroid Imaging Reporting and Data System (TIRADS) guidelines that classify nodules based on visual and textural characteristics. These are composition, shape, size, echogenicity, calcifications, margins, and vascularity. This work aims to reduce subjectivity in the current diagnostic process by using geometric and morphological (G-M) features that represent the visual characteristics of thyroid nodules to provide physicians with decision support. A total of 27 G-M features were extracted from images obtained from an open-access US thyroid nodule image database. 11 significant features in accordance with TIRADS were selected from this global feature set. Each feature was labeled (0 = benign and 1 = malignant) and the performance of the selected features was evaluated using machine learning (ML). G-M features together with ML resulted in the classification of thyroid nodules with a high accuracy, sensitivity and specificity. The results obtained here were compared against state-of the-art methods and perform significantly well in comparison. Furthermore, this method can act as a computer aided diagnostic (CAD) system for physicians by providing them with a validation of the TIRADS visual characteristics used for the classification of thyroid nodules in US images.

## 1. Introduction

The thyroid is one of the largest endocrine glands located below the epiglottis. It is responsible for several physiological functions such as the production of hormones, regulation of brain and nerve cells, and development and functioning of organs like the heart, eyes, hair, skin, and intestines [1]. Irregularities and/or deformations of the thyroid lead to its inability to efficiently carry out these functions. Nodules within the thyroid are mostly benign neoplasms. Ultrasound (US) imaging is typically used as the first point of diagnosis and also for the evaluation of the thyroid nodules, as it effectively images and visualizes soft tissue structures. Additionally, it is free of ionizing radiation and is the most widely available and affordable imaging modality [2]. 

The assessment and evaluation of thyroid nodules using US imaging is done by the physician based on the visual characteristics observed in the scan. For this purpose, the Thyroid Imaging Reporting and Data System (TIRADS) approach is used for the risk stratification and classification of benign vs. malignant nodules. Multiple versions of TIRADS exist such as that of the American Council of Radiology (ACR) TIRADS [3], Kwak-TIRADS [4], etc. Each of these versions differs, but all consider visual and textural features, such as margin, shape, calcification, composition, size, echogenicity and offer a scoring scheme that enables the physician to assess nodule malignancy. 

The use of TIRADS has helped to standardize the evaluation of thyroid nodules found in US images. However, there still exists a considerable amount of inter-observer variability and overall subjectivity. To address the issue of subjective diagnoses several computer aided diagnostic (CAD) methods were proposed. These CAD methods use various feature extraction and classification algorithms to characterize thyroid nodules using US images into benign and malignant. 

The aim of our work was to develop a more objective diagnostic approach for thyroid nodules using US images. The related studies significant to our work will be discussed in the following sub-section.

### Related Work

Several methods have been proposed for feature extraction and computer-aided classification of thyroid nodules using US images. Apart from texture-based features [5], there are other types of feature extraction methods that can be used, like general shape-based feature extraction/classification using different image modalities, as well as shape-based feature extraction for the classification of thyroid nodules in US images. For that we also considered studies that have used the same database that we accessed.

Jianhua Liu et al. introduced the use of shape features of an image. The boundary and region-based feature extraction methods are explained in [6]. The use of shape features was observed in several applications for medical image analysis. Riti et al. employed shape features for the classification of lung cancer from computed tomography (CT) images and obtained an overall classification accuracy of 85% [7]. The same was seen in the work of Ferreira Junior et.al. where the margin sharpness was used for the classification of lung nodules in CT images [8]. Hiremath et al. suggested a shape feature-based approach for detecting follicles in ovarian ultrasound images. The follicles were classified depending on medical knowledge on parametrically defined measures, such as area, compactness, centroid, etc. [9]. Huang et al. in their research suggested the use of functional morphological characteristics to differentiate between benign and malignant breast tumors efficiently. Nineteen morphological features were extracted from ultrasound images and used for the classification of tumors. They obtained a classification accuracy of 82% and a sensitivity of 94% using a support vector machine (SVM) classifier [10]. Nugroho et al. also made use of shape-based feature analysis and extraction for the classification of breast nodules with a specific focus on the marginal characteristics of uncircumscribed versus circumscribed margins [11]. 

In the case of thyroid nodules, Gopinathan et al. performed thyroid nodule risk stratification and classification by analyzing the roundness and irregularity of nodule margins while using US and fine-needle biopsy [12]. Zulfanahri et al. suggested a system that can classify thyroid nodules in two groups, i.e., round to oval and irregular shapes using three chosen characteristics. The suggested system achieved an accuracy of 91.52% and specificity of 91.35% [13]. Similarly, Lina Choridah et al. proposed a technique to classify thyroid nodules based on marginal features. The suggested strategy effectively classified the thyroid nodules into two smooth and uneven classes using US images and obtained an accuracy and sensitivity of 92.30% and 91.88% respectively [14]. The image pattern classification technique used by Junying Chen et al. to categorize benign and malignant thyroid nodules proved efficient in the classification process to verify the types of pattern properties that could be used for the classification of thyroid nodules in US images [15]. 

Ding et al. defined statistical characteristics and texture based on elastography of the thyroid lesion area. The selection of features was then done using the algorithm called minimum redundancy-maximum-relevance. The selected features were then plugged into an SVM classifier [16]. 

Isa et al. used different multi-layer perceptron (MLP) models for the detection of thyroid diseases [17]. Statistical features coupled with demographic details of a sample were fed into three different classification algorithms such as random forest classifier (RFC), SVM, and logistic regression by Patricio et al. to differentiate between thyroid nodules [18]. Song et al. in their work with thyroid nodule US image classification proposed a hybrid multi-branch convolutional neural network (MBCNN) based on a feature cropping method for feature extraction. This work used an open-access dataset [19] as well as a local dataset and obtained a classification accuracy of 96.13% [20]. Chi et al. by using a fine-tuned deep convolutional neural network (FDCNN) approach obtained a classification accuracy of 98.29% [21]. Using the same dataset, Koundal et al. proposed a complete image-driven thyroid nodule detection approach that was able to detect thyroid nodules with an accuracy of up to 93.88% [22]. Nanda and Martha in their work with cancerous thyroid nodule detection employed local binary pattern variants (LBPV)-based feature extraction to classify between benign and malignant thyroid nodules with an accuracy of 94.5% [23] using [19]. 

In the presented literature it can be seen that there are several methods for the feature extraction and classification of thyroid nodules. Studies [6,7,8,9,10,11] give a broad understanding of the use of shape-based features in medical image analyses. Using various combinations of these shape-based features, studies [12,13,14,15] were able to classify thyroid nodules in US images with significant outcomes. Deep learning-based methods [20,21] result in an automatic feature extraction and classification method that differs from handcrafted features. The methods seen in the literature perform well, but most of them do not take into account the geometrical and morphological attributes of thyroid nodules following TIRADS. Since physicians exhibit significant levels of trust in TIRADS, it is essential to consider the features put forth by [3,4]. These are the attributes that are visible to the physicians on which their decision is partly based. Even the studies [12,13,14,15] that do take into account shape features do so only to a limited extent. 

The purpose of this study is to use geometric and morphological feature extraction that takes into consideration features that are closely related to the visual shape-based (TIRADS) features currently used by physicians. This provides them with additional information and mathematical evidence to support their current TIRADS-based classification with an extra layer of objectivity. 

In this work, we mainly focus on shape-based geometric and morphological feature extraction for the classification of thyroid nodules as either benign or malignant. Examining physicians use visual and textural characteristics to classify a nodule. Geometric and morphological features represent the visual aspect. The performance of the features extracted was evaluated using a RFC. The results obtained from the RFC were compared against other feature extraction and classification techniques found in the state of the art that use the same database. Furthermore, our approach was also compared to other shape-based feature extraction and classification methods found in the literature. The rest of the paper is structured as follows: Section 2 details the materials and methods used in this work. Section 3 presents the results and comparisons drawn with other feature extraction and classification methods, and the final Section 4 then provides a conclusion and discusses the next steps. 

## 2. Materials and Methods

**Figure 1:**
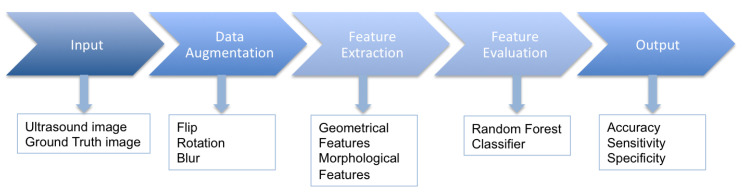
Workflow diagram.

This section is divided into three parts: (1) database and data augmentation, (2) feature extraction and selection, and (3) classification. The section on the database and data augmentation gives details of the dataset used and the various data augmentation techniques used to balance and maximize the data usage. The second section presents details on the geometrical and morphological features extracted from the dataset and discusses the feature selection methods tested and employed for the selection of the most optimal features. The classification section highlights the classification method used to evaluate the selected features with subsequent classification into benign and malignant. The workflow diagram consisting of each step is presented in Figure 1. 

### 2.1. Dataset and Data Augmentation

**Figure 2:**
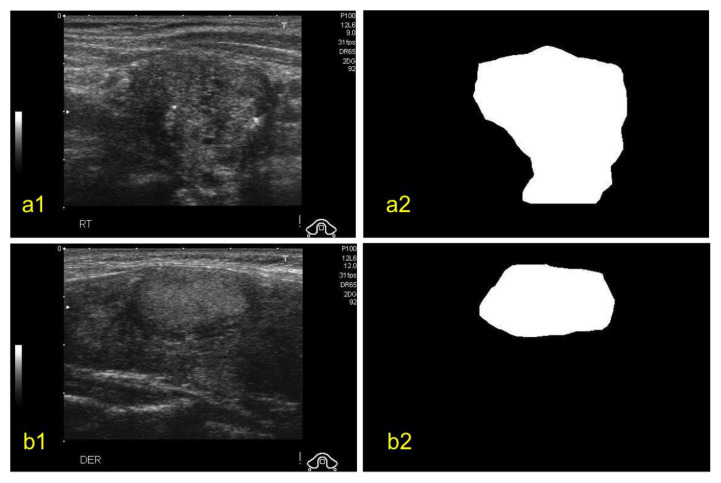
Examples of ultrasound images of thyroid nodules (**a1**) malignant nodule, (**a2**) its ground truth, (**b1**) benign nodule and (**b2**) its ground truth [19].

For this study, we used the Digital Database of Thyroid Ultrasound Images (DDTI) open-access dataset of thyroid nodule ultrasound images from the Instituto de Diagnostico Medico (IDIME) [19]. The dataset consists of a total of 99 cases with 2D ultrasound images from different patients that are annotated and classified based on TIRADS classification. The dataset is divided into JPEG files and XML files. Each image has a resolution of 560 × 315. Each image has a corresponding XML file. The XML files provided a detailed classification for each of the nodules. The ground truth (GT) for each of the nodules was generated by experienced physicians and is available in the form of coordinates in the XML file. An example of benign and malignant nodules along with their GTs can be seen in Figure 2. In this study, we only considered two labels (benign and malignant) rather than all the TIRADS labels.

These cases were divided into 17 benign cases and 82 malignant cases. To correct the data imbalance, data augmentation techniques such as flipping, rotation and blurring were employed. Data augmentation was first used to balance the data and then again to further augment it. Finally, a total of 3188 images were obtained (1594 Malignant + 1594 Benign) from the original 134 images and used in the feature extraction phase of the study. The data augmentation was performed using the Augmentor python package. The percentages of each of the operations (flipping, rotation and blurring) cannot be estimated as they are applied stochastically. 

### 2.2. Feature Extraction

**Figure 3:**
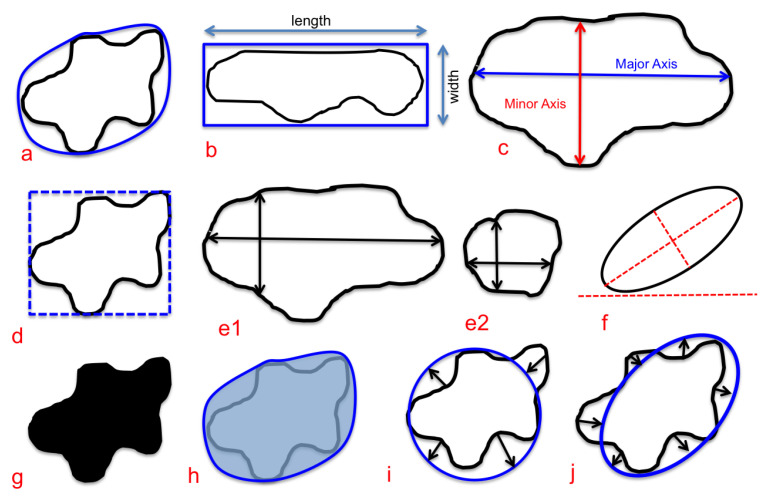
Visual depiction of geometric and morphological features. (**a**) Convexity, (**b**) elongation, (**c**) major and minor axes, (**d**) bounding box, (**e1**,**e2**) different instances of eccentricity, (**f**) orientation, (**g**) filled area, (**h**) convex area, (**i**) circular variance and (**j**) elliptical variance.

In this work, two types of feature were extracted from the GTs of the US thyroid images. These were in the form of geometric and morphological (G-M) features. A summary of all the extracted features can be seen in Table 1. Figure 3 gives a visual depiction of a few geometric and morphological features.

#### 2.2.1. Geometric Features

Geometric features are those features that are used to construct an object with certain geometric elements such as points, curves, and lines as well as information related to edges that describe the shape or irregularity of a given boundary [24,25]. Note that all instances of the mention of the word “object” refer to thyroid nodules. The geometric features extracted from the images are as follows. 

Convex Hull:

The convex hull of an object is the smallest convex structure within which an object is positioned. It is the smallest convex polygon that can contain the object. [26,27]: 

Convexity: 

Convexity is a function that measures the ratio of the convex hull with respect to the original contour of the shape. In this case, a convex hull is drawn around the original contour of the object. Convexity is calculated using the following equation [28]:

(1) $$\mathrm{Convexity}=\frac{P_{c}}{P_{n}}$$

where *P_c_* = perimeter of the convex hull and *P_n_* = perimeter of the object

Solidity: 

Solidity helps describe the extent of a shape’s convexity or concavity. It is given by Equation [29]:

(2) $$\mathrm{Solidity}=\frac{A_{n}}{A_{c}}$$

where *A_n_* = area of the object and *A_c_* = area of the convex hull. A value of a solid object is 1 and an object with an irregular boundary is defined with a value less than 1.

Elongation: 

Elongation is the feature that measures the ratio between the length and width of a bounding box around an object. The result is a nodule’s elongation measurement given as a value of 0 to 1. The object is approximately square or round shaped when the ratio is equal to 1. When the ratio is lower than 1, the object is more elongated. The equation can be seen below [26,30]:

(3) $$\mathrm{Elongation}=\frac{W_{n}}{L_{n}}$$

where *W_n_* = width of the object and *L_n_* = length of the object

Compactness: 

Compactness is the ratio between the areas of an object with respect to the area of a circle with a perimeter equal to that of the object. It is given by the equation below [26,30]:

(4) $$\mathrm{Compactness}=\frac{4\pi A_{n}}{P_{n}^{2}}$$

where *P_n_* = perimeter of the object and *A_n_* = Area of the object 

Rectangularity: 

Rectangularity is defined as the ratio between the object area and the area of the minimum-bounding rectangle [31]. When an object returns a rectangularity of 1 it is said to be a rectangular object.

(5) $$\mathrm{Rectangularity}=\frac{A_{n}}{A_{r}}$$

where *A_n_* = area of the object and *A_r_* = Area of the rectangle.

Roundness: 

The roundness of an object is defined as the ratio between the area of the object and the area of a circle with the same convex perimeter. It can be represented by the following equation [26,32]:

(6) $$\mathrm{Roundness}=\frac{4\pi A_{n}}{P_{c}^{2}}$$

where *P_c_* = the convex perimeter of the object and *A_n_* = the area of the object

Major Axis length: 

The major axis of an object is the endpoints (*X*, *Y*) of the longest line traced through the object. The endpoints of the major axis (*X*1, *Y*1, and *X*2, *Y*2) are determined by calculating the pixel distance between every combination of border pixels in the object boundary. This is used to find the pair with the maximum length. The object’s major axis length is the pixel distance between the major axis endpoints and is defined by the equation [26,32]:

(7) $$\text{Major-axis length}=\sqrt{{\left(X2-X1\right)}^{2}+{\left(Y2-Y1\right)}^{2}}$$

Minor Axis length:

The minor axis is the (*x*, *y*) endpoints of the longest line drawn by the object while still perpendicular to the major axis. The endpoints (*x*1, *y*1, and *x*2, *y*2) of the minor axis are calculated by computing the pixel distance between the two border pixel endpoints. The minor-axis length of an object is the pixel distance between the minor axis endpoints and is defined by the equation [26,32]:

(8) $$\text{Minor-axis length}=\sqrt{{\left(x2-x1\right)}^{2}+{\left(y2-y1\right)}^{2}}$$

Eccentricity: 

Eccentricity is defined as the ratio between minor axis length and the major axis length of the object [26]. The result is a measure of the eccentricity of the object, given as a value from 0 to 1.

(9) $$\mathrm{Eccentricity}=\frac{L_{ma}}{L_{MA}}$$

where *L_ma_* = length of minor axis and *L_MA_* = Length of major axis 

Circular Variance: 

Circular variance (C_va_) is the comparison of an object’s shape with respect to a known shape such as a circle. The circular variance is the object’s proportional mean-squared error with respect to the solid circle [26,32]. This returns a null value for a perfect circle and increases and the shape and complexity change. It is given by the following equation.

(10) $$C_{\mathrm{va}}=\frac{\sigma R}{\mu R}$$

where *µR* and *σR* are the mean and standard deviation of the radial distance from the centroid (*c_x_*, *c_y_*) of the shape to the boundary points (*x_i_*, *y_i_*), *i* ϵ [0, *N* − 1]. 

They are represented by the formulae:

(11) $$\mu R=\frac{1}{N}\overset{N-1}{\underset{i=1}{\sum }}d_{i}$$

and

(12) $$\sigma R=\sqrt{\frac{1}{N}\overset{N-1}{\underset{i=1}{\sum }}{\left(d_{i}-\mu R\right)}^{2}}$$

where $d_{i}$ = $\sqrt{{\left(x_{i}-c_{x}\right)}^{2}+{\left(y_{i}-c_{y}\right)}^{2}}$

Elliptic Variance:

Elliptic variance (E_va_) is measured similarly to the circular variance. An oval is fitted to the shape (rather than a circle), and the mean squared error is estimated [32].

(13) $$E_{\mathrm{va}}=\frac{\sigma^{\prime }R}{\mu^{\prime }R}$$

(14) $$\mu^{\prime }R=\frac{1}{N}\overset{N-1}{\underset{i=1}{\sum }}{d^{\prime }}_{i}$$

and

(15) $$\sigma^{\prime }R=\sqrt{\frac{1}{N}\overset{N-1}{\underset{i=1}{\sum }}{\left({d^{\prime }}_{i}-\mu^{\prime }R\right)}^{2}}$$

where

$d\prime_{i}=\sqrt{V_{i}^{T}\times C_{ellipse}^{-1}\times V_{i}}$
$C_{ellipse}^{-1}$ = Inverse of covariance matrix of the shape (ellipse)
$V_{i}=\left(\frac{x_{i}-c_{x}}{y_{i}-c_{y}}\right)$ and $V_{i}^{T}=\mathrm{Transpose}\;\mathrm{of}\;V_{i}$

Ratio of Major Axis Length to Minor Axis Length:

Thus is the ratio between major axis lengths to minor axis length.

(16) $$\mathrm{Ratio}\;\mathrm{of}\;\mathrm{length}\;\mathrm{of}\;\mathrm{Major}\;\mathrm{axis}\;\mathrm{and}\;\mathrm{minor}\;\mathrm{axis}\;=\;\frac{L_{MA}}{L_{ma}}$$

where *L_MA_* = length of major axis and *L_ma_* = length of minor axis.

Orientation: 

The orientation is angle between the x-axis and the major axis of the object. It can also be defined as the direction of the shape [26].

Bounding Box: 

The bounding box is the region’s smallest rectangle that envelops the object [25]. Dimensions for the bounding box are those equal to the major and the minor axes.

(17) $$\mathrm{Area}\;\mathrm{of}\;\mathrm{bounding}\;\mathrm{box}\;=\;L_{MA}\times L_{ma}$$

where *L_MA_* = length of major axis and *L_ma_* = length of minor axis.

Centroid: 

The centroid is defined as the center of gravity of the object [25].

Convex Area: 

The convex area of a nodule is the area surrounded by the convex hull [31].

Filled Area: 

Is the total number of pixels within the marked object mask. [26]

Convex Perimeter: 

The convex perimeter of an object is the perimeter of the convex hull that encloses the object [25].

#### 2.2.2. Morphological Features

Morphological features are those features that define an object’s structuring elements such as area, perimeter, aspect ratio, etc. [33]. The following morphological features were considered for the classification of thyroid nodules in this work.

Area: 

Area is the space occupied by objects on a plane surface. Here area is defined as the number of pixels inside the object region [34].

Perimeter: 

The number of pixels within the object border is the perimeter [26,34]. If *x1… xN* is a list of boundaries, and the perimeter is defined by:

(18) $$\mathrm{Perimeter}=\sum_{i=1}^{N-1}di=\sum_{i=1}^{N-1}\left|X_{i}-X_{i+1}\right|$$

Aspect Ratio: 

The aspect ratio is defined as the ratio between the tumor’s depth and width [26]:

(19) $$\mathrm{Aspect}\;\mathrm{Ratio}=\frac{D_{n}}{W_{n}}$$

where *D_n_* = Depth of the object and *W_n_* = Width of the object.

AP Ratio (area to perimeter (AP) ratio): 

The AP ratio is the ratio between object area and perimeter of the object, and it is defined as:

(20) $$\mathrm{AP}\;\mathrm{Ratio}=\frac{A_{n}}{P_{n}}$$

where *A_n_* = Area of the object and *P_n_* = Perimeter of the object 

TEP Ratio (object perimeter to ellipse perimeter ratio): 

The TEP ratio is the ratio of perimeters of an object to the related ellipse [17].

(21) $$\mathrm{TEP}\;\mathrm{Ratio}=\frac{P_{n}}{P_{e}}$$

where *P_n_* = perimeter of the object and *P_e_* = perimeter of the ellipse

TEP Difference:

TEP is determined by the difference between the object perimeter and the related ellipse [17]:

TCP Ratio (object perimeter to circle perimeter ratio): 

The TCP ratio is the ratio of the perimeter of the object to the relevant circle [17]:

(22) $$\mathrm{TCP}\;\mathrm{Ratio}=\frac{P_{n}}{P_{c}}$$

where *P_n_* = perimeter of the object and *P_c_* = perimeter of the circle. 

TCP Difference:

The TCP difference is known as the difference between the perimeter of the object and the corresponding circle [17]. 

#### 2.2.3. Feature Selection

Feature selection was based on the most relevant visual characteristics in accordance with TIRADS. This was determined with the help of the two physicians in the Department of Nuclear Medicine at the University Hospital in Magdeburg, Germany. Clinicians use TIRADS classification that classifies a nodule based on its geometrical attributes such as shape, size, irregularity in margins and orientation. The 11 selected features provide the closest estimation to these attributes used by TIRADS. In order to further validate this claim the authors tested the performance of all the geometric and morphological features (Global) as well as those that were not selected (discounted). The performance metrics for each of these are seen in Table 4 in Section 3. 

Labels were added to each row of features depending on whether they belonged to 0 = benign or 1 = malignant. An overview of all the extracted features can be seen in Table 1. The feature extraction process was carried out using MATLAB 2018b. The feature sets were then exported as .csv files and used for the classification process. The performance of the features was evaluated using a RFC. 

### 2.3. Classification

An RFC was trained for a binary classification problem where each of the feature rows was labeled as 0 or 1. RFC is a type of ensemble learning that builds a final classifier by using weak individual classifiers i.e., binary decision trees. Each tree is a collection of nodes and features that lead to the final classification result. The aggregation of the results from each of the individual trees is considered the final result of the classifier. The features were used as the independent variables and the labels as the dependent variables. The parameters chosen for the RFC are shown in Table 2. A train-test split of 70–30% was used on the data. The classification using RFC was undertaken in Python 3.7 using the scikit-learn library. 

## 3. Results and Discussion

A total of 27 features were extracted from the thyroid nodule dataset. The feature selection step led to the selection of the 11 most significant features. We selected three prime metrics to compare the G-M features against the performance of global and discounted features in our study for the feature selection as well as the methods found in the literature. The metrics selected were accuracy, sensitivity, and specificity.

The 11 significant features are given in Table 3. These features were selected based on clinical input and expertise. Further experiments confirmed the validity of the features selected. This is highlighted in Table 4. 

As can be seen the accuracy scores of global features and discounted features are considerably lower (70.18% and 61.55% respectively) than compared to G-M. Additionally, both global and discounted features exhibit high specificities, but the sensitivities were low (48.07% and 31.65% respectively). As this is a case of cancer classification, more focus was given to the sensitivity score due to its clinical relevance (true positive rates). The selected 11 final features resulted in high accuracy, specificity and sensitivity scores. i.e., the classifier was able to identify benignity and malignancy of the nodules much more effectively. 

The selected 11 features were fed into the RFC and the results obtained were compared against the results from [20,21,23] that used deep learning-based and LBPV-based methods. These studies used feature extraction and classification techniques that were different from our proposed approach but were all tested on the same open-access dataset [19]. Additionally, the results were further compared against other shape-based feature extraction techniques [13,14] found in the literature. 

The same performance metrics were used to present the results obtained from the classifiers. This is presented in Table 5 and Table 6 below. The comparison is done in two steps. The first step involves the comparison between the proposed method and the methods found in the state of the art that used the same dataset as us. The second step draws a comparison between the proposed method and other thyroid nodule feature extraction and classification approaches using shape-based features found in other related studies. 

Table 5 shows the classification results obtained from our proposed feature extraction approach, G-M and three different thyroid nodule feature extraction and classification approaches found in the state of the art. i.e., MBCNN [20], FDCNN [21] and LBPV [23]. Each of these methods uses a different feature extraction and classification method from the proposed approach. However, the dataset used in all four cases is the same [19]. Data augmentation was used by [20,21]. It can be seen from the table that G-M based features display a higher accuracy of 99.33% as compared to [20,21,23] while using the same database. G-M features also result in a high specificity score of 99.25%. In the case of sensitivity, G-M features exhibit a score of 99.39%. From the depicted results it can be inferred that the proposed feature extraction method can classify benign and malignant thyroid nodules with high accuracy. Additionally, this approach also displays high true positive (Sensitivity) and true negative (Specificity) rates. This means that the feature extracted can be distinguished well.

Table 6 shows the classification results obtained from the proposed G-M feature extraction method compared to two approaches found in the literature that use shape-based features for the classification of thyroid nodules. Each of the two studies [13,14] compared to G-M uses features that help characterize the nodules based on the extent of the margins being oval. Both the compared methods use different datasets in their work. It can be seen from the table that the G-M features when fed into the RFC classifier exhibit an accuracy, sensitivity, and specificity of 99.33%, 99.39%, and 99.25%, respectively. This is a considerable improvement over the same metrics seen in [13,14]. 

In both comparison cases, across different feature extraction methods using the same dataset as well as similar feature extraction methods using different input data, the G-M approach outperforms the state-of-the-art. Even though [13,14,20,21,23] show high accuracies, sensitivities, and specificities, the features extracted are often not relevant for the physician performing the examination. To a certain extent, [13,14] take into account some shape features. But, [20,21,23] use features that are not in accordance with TIRADS. While reviewing US images of thyroid nodules physicians take into consideration the visual and textural characteristics of a nodule to classify it as benign or malignant. The geometric and morphological features encompass the visual characteristics of a nodule. These are the closest estimation to the features defined by the gold standard in TIRADS. This can be observed across all three calculated metrics. It is evident that the G-M features adequately emulate the visual characteristics that are defined in TIRADS. These visual aspects of a thyroid nodule such as margins irregularity and shape can be directly attributed to the G-M features extracted in this work. 

## 4. Conclusions and Future Work

This study focused on the geometric and morphological feature extraction techniques for the classification of thyroid nodules from US images. This work used a total of 3188 images. A total of 27 geometric and morphological features were defined and extracted from these images and then the 11 most significant features selected in accordance with the TIRADS-based visual features and labeled based on their class (0 = benign, 1 = malignant). The performance of the selected features was then evaluated by the classification accuracy, sensitivity, and specificity obtained from the RFC. 

The consideration of G-M features for a computer-aided diagnostic approach for thyroid nodules is clinically relevant. The 11 selected features in this study proved to be the best combination from the overall feature set of 27 because these selected features provide information such as the shape, irregularity in the boundary, orientation, and size of the US thyroid nodule. However, it must be noted that G-M features are only one part of the features that help in the classification. According to TIRADS, physicians also need to consider texture features found in a nodule, which were not considered for this work. Hence, a notable observation of the proposed approach is that it only considers one classification aspect. When G-M features are combined with texture features the overall accuracy might change, which needs to be studied further. Another limitation of this work is that everything was carried out on a single open-source database and further validation of the approach needs to be carried using additional datasets including different ultrasound scanners. We are currently working on acquiring data at the Department of Nuclear Medicine at the University Hospital in Magdeburg, Germany. This would help to improve the robustness of the features extracted across different datasets. 

In future work, we would like to omit the use of data augmentation techniques all together. However, this is dependent on the amount of data that is being currently collected as mentioned. Until large volumes of US thyroid nodule images are available, there are two possible strategies that can be employed additionally to test the relevance of the selected features. The first is to augment the data while being aware of the percentage of each operation (flipping, rotation and blurring) performed. This would help us determine and understand how each of the selected features behaves with respect to each data augmentation operation. Additionally, it would also help us determine the extent to which the data augmentation effects the final classification. The second method would be to test the features extracted from non-augmented images against features extracted from augmented ones and to show the deviation between these two sets. 

The addition of texture-based features to the G-M features would provide a larger feature set that would then consider both aspects of a TIRADS classification and provide physicians with better decision support for the classification of thyroid nodules. Hence, a step towards reducing inter-observer variability and overall diagnostic subjectivity. 

## Figures and Tables

**Table 1:** Overview of 27 extracted geometric and morphological (G-M) features.

Sr. No.	Features	Type
1	Convex Hull	**Geometric Features**
2	Convexity	
3	Solidity	
4	Elongation	
5	Compactness	
6	Rectangularity	
7	Orientation	
8	Roundness	
9	Major Axis Length	
10	Minor Axis Length	
11	Eccentricity	
12	Circular Variance	
13	Elliptic Variance	
14	Ratio of Major Axis Length to Minor Axis Length	
15	Bounding Box	
16	Centroid	
17	Convex Area	
18	Filled Area	
19	Convex Perimeter	
20	Area	**Morphological Features**
21	Perimeter	
22	Aspect Ratio	
23	Area Perimeter (AP)Ratio	
24	Object Perimeter to Ellipse Perimeter (TEP) Ratio	
25	TEP Difference	
26	Object Perimeter to Circular Perimeter (TCP) Ratio	
27	TCP Difference	

**Table 2:** Selected random forest classifier (RFC) parameters.

Parameter	Value
Number of Decision Trees	400
Criterion	Entropy
Bootstrap	True

**Table 3:** Eleven most significant features selected from the global feature list of 27.

Sr. No.	Features	Type
1	Solidity	**Geometric Features**
2	Orientation	
3	Roundness	
4	Major Axis Length	
5	Minor Axis Length	
6	Bounding Box	
7	Convex Area	
8	Area	**Morphological Features**
9	Perimeter	
10	Aspect Ratio	
11	AP Ratio	

**Table 4:** Performance metrics of selected features versus global and discounted features.

Method	Accuracy (%)	Sensitivity (%)	Specificity (%)
Global	70.18	48.07	92.29
Discounted	61.55	31.65	91.45
G-M	99.33	99.39	99.25

**Table 5:** Feature evaluation using RFC compared to the performance of the related approaches using the same dataset.

Method	Accuracy (%)	Sensitivity (%)	Specificity (%)
MBCNN [20]	96.13	97.18	-
FDCNN [21]	98.29	99.10	93.90
LBPV (SVM) [23]	94.5	97.25	94.50
G-M (RFC)	99.33	99.39	99.25

**Table 6:** Feature evaluation using RFC compared to the performance of shape-based features found in related studies using different datasets.

Method	Accuracy (%)	Sensitivity (%)	Specificity (%)
Margin Features [13]	91.52	91.80	91.35
Margin Features [14]	92.30	91.88	92.73
G-M	99.33	99.39	99.25
